# How many to sample? Statistical guidelines for monitoring animal welfare outcomes

**DOI:** 10.1371/journal.pone.0211417

**Published:** 2019-01-30

**Authors:** Jordan O. Hampton, Darryl I. MacKenzie, David M. Forsyth

**Affiliations:** 1 Murdoch University, Murdoch, Western Australia, Australia; 2 Ecotone Wildlife Veterinary Services, Inverloch, Victoria, Australia; 3 Proteus, Outram, New Zealand; 4 Department of Primary Industries, New South Wales Government, Orange, New South Wales, Australia; 5 School of Biological, Earth and Environmental Sciences, University of New South Wales, Sydney, New South Wales, Australia; Universitat Autonoma de Barcelona, SPAIN

## Abstract

There is increasing scrutiny of the animal welfare impacts of all animal use activities, including agriculture, the keeping of companion animals, racing and entertainment, research and laboratory use, and wildlife management programs. A common objective of animal welfare monitoring is to quantify the frequency of adverse animal events (e.g., injuries or mortalities). The frequency of such events can be used to provide pass/fail grades for animal use activities relative to a defined threshold and to identify areas for improvement through research. A critical question in these situations is how many animals should be sampled? There are, however, few guidelines available for data collection or analysis, and consequently sample sizes can be highly variable. To address this question, we first evaluated the effect of sample size on precision and statistical power in reporting the frequency of adverse animal welfare outcomes. We next used these findings to assess the precision of published animal welfare investigations for a range of contentious animal use activities, including livestock transport, horse racing, and wildlife harvesting and capture. Finally, we evaluated the sample sizes required for comparing observed outcomes with specified standards through hypothesis testing. Our simulations revealed that the sample sizes required for reasonable levels of precision (i.e., proportional distance to the upper confidence interval limit (*δ*) of ≤ 0.50) are greater than those that have been commonly used for animal welfare assessments (i.e., >300). Larger sample sizes are required for adverse events with low frequency (i.e., <5%). For comparison with a required threshold standard, even larger samples sizes are required. We present guidelines, and an online calculator, for minimum sample sizes for use in future animal welfare assessments of animal management and research programs.

## Introduction

There is increasing scrutiny of the animal welfare outcomes of animal use activities, including agriculture, the keeping of companion animals, racing and entertainment, research and laboratory use, and wildlife management programs [1]. Animal welfare is a young science [2] and, while containing philosophical elements necessitating qualitative and discussive studies, it does not have a strong statistical underpinning relative to other life sciences [3]. This weakness can hinder the robustness of efforts to provide regulatory oversight for aspects of animal welfare that are of societal concern. Research activities have oversight from institutional committees (e.g., Animal Ethics Committees; AECs), but there is often little monitoring of outcomes for operational activities [4]. The absence of statistical guidelines for collecting and analysing animal welfare data has led to intractable contention surrounding efforts to monitor industries such as sea transport ‘live export’ of livestock [5–8].

Due to the cost and inconvenience of animal-based monitoring and its resultant unpopularity with operational staff, desktop approaches (e.g., expert opinion) have commonly been used to address societal animal welfare concerns [9]. Desktop animal welfare assessments often make the assumption that ‘best practice’ inputs are followed, as described by procedural documents [9], leading to optimal outcomes being uniformly achieved [10]. Many authors have understandably questioned how often these optimal outcomes are truly achieved in contentious wildlife management programs. For example, a review of management techniques applied to hyperabundant kangaroos (*Macropus* spp.) in Australia questioned how often kangaroos are rendered immediately insensible via accurate head shots [11], as described in procedural documents [12, 13].

One approach to answering questions about how commonly a desirable animal welfare outcomes occur is to use animal-based measures to quantify the frequency of adverse animal welfare events [4], as is often used by AECs [14]. Adverse animal welfare events must be those that are easily identified and whose classification is not controversial. For example, mortalities for transported livestock [6], injuries in racing animals [15], non-fatal wounding for harvesting wild game [16], or escapes in wildlife capture programs [17] are straightforward to report. From an ethics perspective, quantifying the frequency of adverse events gives priority to the animals that are likely to be worst off [18], not necessarily solely looking at the total aggregated welfare outcome [19]. This approach has been used for decades in audits of livestock slaughter through reporting of parameters such as the proportion of cattle that are not rendered immediately insensible via stunning [20–22]. Monitoring of adverse event data is also used in veterinary medicine to assess the safety and efficacy of registered therapeutic chemicals [23] and anaesthetic procedures [24]. Reporting such data allows identification of techniques that produce favourable animal welfare outcomes when compared with those that do not and can facilitate the development of evidence-based ‘best practice’ guidelines [25].

Monitoring the frequency of adverse events can also be applied to regulation, with results providing pass/fail grades for management programs relative to a threshold specified by procedural documents. This approach is referred to as the use of ‘animal welfare standards’ [9, 26]. For example, for sea transport of sheep from Australia, until recently there was a requirement for the mortality rate to be <2% [5, 27]; for slaughter of cattle in some assurance schemes, there is a requirement that the frequency of non-immediate insensibility be <5% for captive-bolt euthanasia [22]. However, there are no published guidelines available for the data collection necessary to quantify such parameters. Without robust statistical approaches, such important questions can only be answered speculatively. As a consequence, sample sizes in animal welfare audits of operational activities have been highly variable, ranging from 10 animals [28] to >100,000 animals [6]. This variability has led to uncertainty about the reliability of the conclusions of these audits. For example, would an assessment failing to detect non-fatal wounding in 28 of 28 animals exposed to a wildlife shooting program be sufficient to conclude that 100% of animals experience optimal outcomes [29]? On the other hand, would an assessment detecting desirable outcomes in 27 of 28 (96%) animals be sufficient to conclude that the program outcomes exceed an animal welfare standard of 95% [30]?

In this study, first, we used statistical theory to calculate the sample size required for determining the frequency of an adverse event (typical of an animal welfare study) at different levels of precision. Second, we assessed a broad selection of published animal welfare studies to examine the confidence intervals of the frequency parameters they reported. Third, we elucidated the relationship between the number of animals sampled and the statistical ability to determine whether an animal use activity exceeded or failed the required threshold standard for any designated parameter. Fourth, we tested the statistical power of a variety of published studies in order to determine whether they exceeded or failed relative to a hypothetical animal welfare standard.

## Materials and methods

### Statistical precision of estimates from sample surveys

Aspects of statistical theory may be familiar to readers working in fields such as ecology, epidemiology and animal production. However, these theoretical considerations may be less familiar to animal welfare scientists. We therefore provide a brief summary of the key elements of statistical theory related to outcome monitoring.

**Fig 1:**
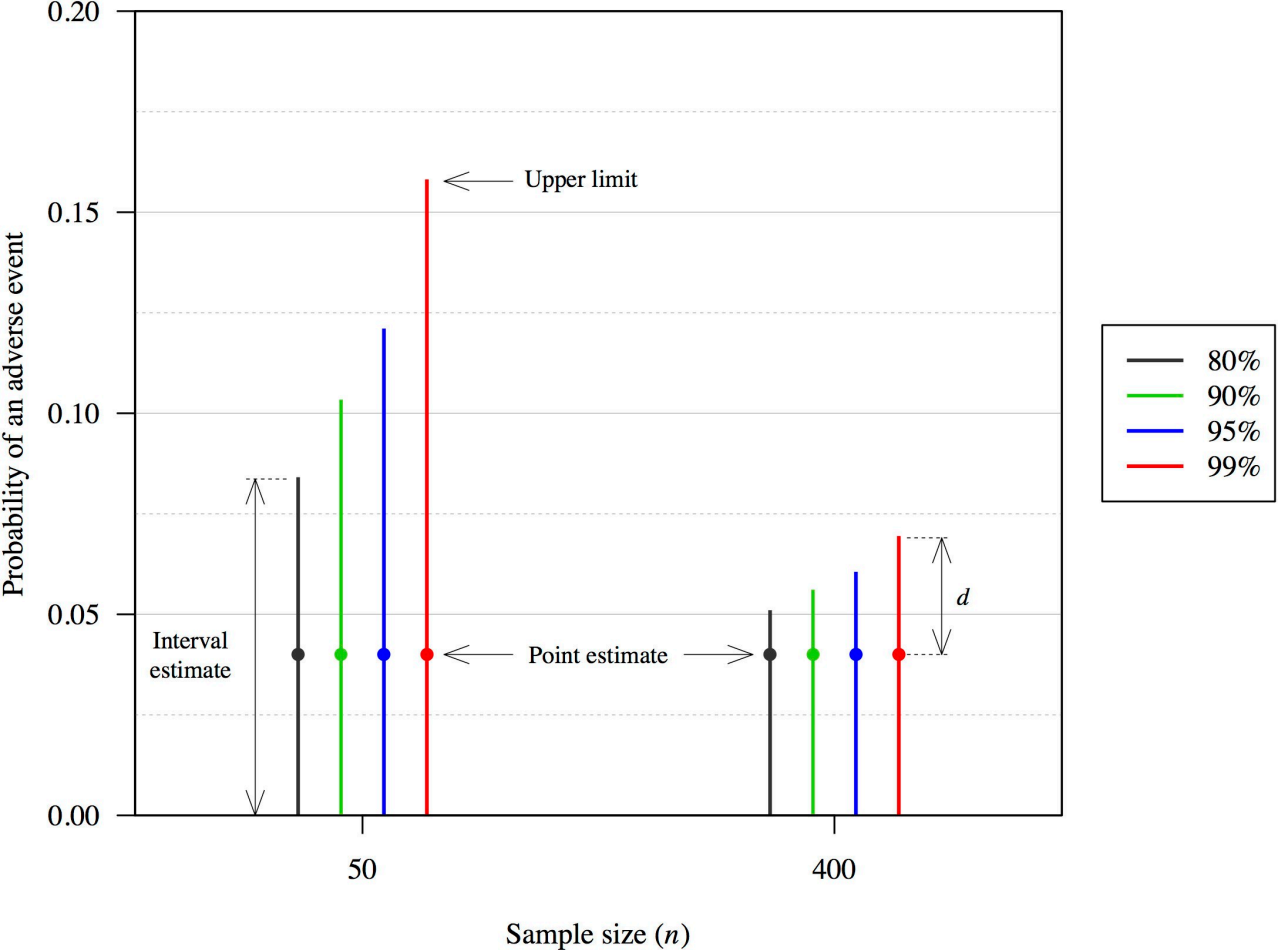
Examples of one-sided confidence interval estimates of frequency, with a point estimate of 0.04. Confidence levels of 80% (black), 90% (green), 95% (red) and 99% (blue), and sample sizes (*n*) of 50 and 400 are presented to illustrate the effect on the resulting interval estimate. The distance from the point estimate to the upper limit is indicated (*d*).

At the most basic level, animal welfare outcome monitoring constitutes incomplete sampling of a population, because not all individuals are checked to assess their welfare outcome. Extrapolation to the larger population is attempted from sampled data, but needs to account for uncertainty due to the incomplete sampling. Multiple metrics can be used to express the uncertainty in the point estimate of a quantity of interest (e.g., proportion of individuals with an adverse outcome), including standard error (SE) and coefficient of variation (CV). Alternatively, an interval estimator can be used that expressly incorporates the uncertainty into a calculated estimate (Fig 1). A confidence interval estimate is one such estimate. A confidence interval estimate can be one-sided or two-sided. For a two-sided interval, the values of the upper and lower limits are calculated to define the range of the interval estimate [31]. For a one-sided interval, the value of either the upper or lower limit is calculated, and the other limit is set at the smallest or largest possible value for the parameter. A one-sided limit is arguably more appropriate in animal welfare settings in which operational protocols stipulate that the probability of an adverse event must be less than a threshold value.

The range of a confidence interval estimate is affected by two things: the desired confidence level and the uncertainty in the quantity of interest (Fig 1). Uncertainty is determined by both the degree of variation in the observations and the sample size. The confidence level is a theoretical property of the interval estimator being used, and it relates to the frequency with which the interval would encompass the unknown true value of the quantity of interest for the population if it were possible to repeatedly resample the population [31]. A higher level of confidence leads to a wider interval (Fig 1), as a wider interval is more likely to encompass the true (but unknown) value. We stress that the confidence level is a theoretical property of the method used to calculate an interval estimate, and defines the type of interval estimate being calculated.

For a given set of data, it is unknowable whether the interval estimate encompasses the true value or not, and one should not attempt to draw conclusions in this regard (although this is commonly done). The interval is simply a particular type of estimate of the quantity of interest, expressed as a range of values rather than a single value (i.e., a point estimate). Typically, it is desirable to use a high confidence level, and 95% is commonly specified [32].

In some applications, the precision of an estimate may be expressed as the ‘margin of error’ [32]. The margin of error is a statistic expressing the amount of random sampling error in a study’s results, and is half of the total range of a symmetric two-sided confidence interval, that is, the distance either side of the point estimate to the upper and lower limits of the confidence interval. For example, if the estimated frequency of an individual animal being in a capture program is 30% with a confidence interval estimate of 27–33%, the margin of error is ±3%.

### Sample size calculation for estimation of frequency

We assumed that *N* animals were involved in an operational activity (day-to-day use of animals, as opposed to research), of which *n* were sampled and assessed for an adverse welfare outcome. The case being considered here was for situations in which *n* was small relative to *N*, such that a small percentage of the population of interest was sampled (e.g., <10%). If *p* is the probability of an individual suffering an adverse outcome, or the frequency of an adverse outcome in the population, then the number of adverse outcomes in an operational activity (*x*) would be expected to follow a binomial distribution. A variety of methods can be used to obtain confidence interval estimates for *p*. Some methods are based on using the properties of the normal distribution to approximate a binomial distribution, which works well provided *n* is large relative to *p*, and *p* is not close to 0 or 1 [33]. Alternatively, so-called ‘exact methods’ can be used that are based on the properties of the binomial distribution; these methods are more accurate when *n* is smaller or *p* is close to 0 or 1, as could be expected when estimating the probability of an animal experiencing an adverse event, which would usually be low [31].

One such ‘exact method’ is the Clopper–Pearson method [34], which can be used to estimate either a one-sided or two-sided confidence interval. Confidence intervals estimated using the Clopper-Pearson method always has coverage probability of at least 1 –α for every possible value of *p*. Some statisticians consider the Clopper-Pearson method to be overly conservative, with the actual coverage probability much larger than the nominal confidence interval unless *n* is large e.g., [35]. Numerous alternative methods have been recommended, including the Wilson method (‘score confidence interval’) [35]. Following an extensive comparison of the Clopper–Pearson exact and the Wilson methods (S1 Appendix) we chose to use the former here, but we emphasise that the Clopper–Pearson exact is a conservative method and that multiple other methods could be used e.g., [35, 36].

In the following development, we assume that *p* will be near 0, but note that the same logic applies when *p* is close to 1, because one can simply reverse the estimation problem from estimating the probability of an animal having an adverse outcome (*p*), to the probability of an animal not having an adverse outcome (p* = 1 –*p*). Hence, when *p* is close to 1, p* will be close to 0. Lower and upper limits for p* then become the upper and lower bounds, respectively, for *p*.

For animal welfare outcome monitoring, the question of interest will primarily be what is the upper bound of the interval estimate. Hence, one-sided intervals are appropriate. The upper bound (${\hat{p}}_{U}$) can be estimated as:

$${\hat{p}}_{U}=\hat{p}+d,$$ (1)

where $\hat{p}$ is the estimated probability (= *x*/*n*) and *d* is the distance to the upper bound. For the purpose of sample size determination, it has been shown that the expected distance to the upper bound (*E*(*d*)) of a (1 –*α*) × 100% confidence interval estimate can be approximated [37] as:

$$E(d)=z_{\alpha }\sqrt{\frac{p(1-p)}{n}}+\frac{z_{\alpha }^{2}(1-2p)+2-p}{3n}$$

where *z*_*α*_ is the value that equates to the (1 –*α*) percentile of the standard normal distribution, for example, *z*_*α*_ = 1.282 for a one-sided 90% confidence interval estimate, and *z*_*α*_ = 1.645 for a one-sided 95% confidence interval estimate.

Eq (2) can be rearranged to determine the sample size required such that the upper limit of a (1 –*α*) × 100% confidence interval estimate is distance *d* from the anticipated probability of the adverse outcome (S2 Appendix). This allows *n* to be determined for specific values of *p*, *α* and *d*. However, *d* is the absolute distance between the value of the upper limit and *p*, and the connotations of a specific value for *d* differ depending on the value of *p*. For example, when *p* = 0.10, a distance of 0.05 from the upper limit suggests a relatively precise estimate, whereas a distance of 0.05 suggests a highly imprecise estimate if *p* = 0.01. It could therefore be more useful to specify the required distance relative to *p*; for example, what sample size is required such that the upper limit is expected to be half as large as the probability of the adverse outcome? That is, let *d* = *δp*, where *δ* is the proportional distance to the upper limit.

### Assessment of confidence intervals in past animal welfare studies

We assessed the precision of six published animal welfare studies that reported the frequency of adverse events in operational activities (Table 1). We also assessed the precision of six published animal welfare studies that reported the frequency of adverse events in research trials (Table 2). These studies were subjectively selected to represent a broad range of animal use activities, countries, species, and sample sizes. The datasets estimated the following frequency parameters: (i) mortality, (ii) injury, (iii) non-immediate insensibility, (iv) hyperthermia, and (v) escape. Exact one-sided confidence interval estimates were calculated for the frequencies of each of these adverse outcomes. Confidence levels of 80%, 90%, 95% and 99% were used to illustrate the methods, although in practice we recommend only one confidence level be used (and defined *a priori*).

**Table 1:** Selected published studies reporting the frequency of adverse animal welfare events from operational activities (not research trials).

Animal species	Operational activity	Adverse event type	Number of adverse events	*n* (sample size)	Source
Domestic horse (*Equus caballus*)	Racing	Injury	2,358	222,993	[15]
Badger (*Meles meles*)	Trapping	Injury	3,015	18,596	[38]
Domestic cattle (*Bos taurus*)	Dairy calf transport	Weak or recumbent animals	27	7,169	[39]
White-tailed deer (*Odocoileus virginianus*)	Helicopter net gunning	Injury	281	3,350	[40]
Impala (*Aepyceros melampus*)	Night shooting	Non-immediate insensibility	54	856	[41]
Caribou (*Rangifer tarandus*)	Helicopter darting	Mortality	12	296	[42]

Animal species, point estimates of frequency, sample sizes, and citations are listed. Studies are listed in descending order of sample size.

**Table 2:** Selected published studies reporting the frequency of adverse animal welfare events from research trials (not operational activities).

Animal species	Research activity	Adverse event type	Number of adverse events	*n* (sample size)	Source
New Zealand fur seal (*Arctocephalus forsteri*)	Ground darting	Escape	16	120	[17]
White-tailed deer (*Odocoileus virginianus*)	Ground darting	Escape	11	23	[43]
Vicuña (*Vicugna vicugna*)	Mustering and transport	Laceration injury	2	19	[44]
Wild horse (*Equus caballus*)	Helicopter darting	Hyperthermia	2	11	[28]
Domestic chicken (*Gallus gallus domesticus*)	Captive bolt	Non-immediate insensibility	0	10	[45]
Green sea turtle (*Chelonia mydas*)	Euthanasia	Non-immediate insensibility	0	2	[46]

Animal species, point estimates of frequency, sample sizes and citations are listed. Studies are listed in descending order of sample size.

### Hypothesis tests

An alternative to estimating the magnitude of the adverse outcome probability with a specified precision is to conduct a statistical hypothesis test for whether the probability is less than a specified value of interest (*p*_*S*_). That is, define the null hypothesis (*H*_*0*_) and alternative hypothesis (*H*_*A*_) as:

$$H_{0}:p\geq p_{S},\;\mathrm{and}$$

$$H_{A}:p<p_{S}$$

The amount of evidence against the null hypothesis can then be evaluated using an exact (one-sided) test for a binomial proportion [47]. Note that the hypotheses are defined such that the ‘burden of proof’ is on demonstrating that the frequency of an adverse outcome is less than the specified value.

In a hypothesis-testing framework, there are two types of ‘errors’ that can be made from the testing procedure: (1) rejecting *H*_*0*_ when *H*_*0*_ is true; and (2) accepting *H*_*0*_ when *H*_*A*_ is true. These are termed Type I and Type II errors, respectively. The probability of a Type I error (also known as the *α*-level) is controlled by the point at which the *p*-value from a testing procedure is declared ‘significant’ and *H*_*0*_ is rejected. The statistical ‘power’ of the testing procedure is the probability that *H*_*0*_ is rejected when *H*_*A*_ is true, that is, the opposite of the probability of a Type II error, which is affected by the *α*-level that is used. In other words, a test’s power is greater when a higher *α*-level is used, but the cost is an increased chance of a Type I error. Ideally, a study should be designed and a hypothesis test used such that there is a low probability of a Type I error and relatively high power (i.e., ≥80%).

The power of the exact test, assuming a binomial distribution, can be calculated for a given *α*-level and sample size, with a true adverse outcome probability, *p*_*T*_, and a specified probability of interest, *p*_*S*_. However, because of the discrete nature of the binomial distribution, it might not be possible to design a study such that it will have the exact *α*-level required. Therefore, often a study will need to be designed so that the actual *α*-level for the exact test will be less than the required value. It is also possible to reverse the design problem: to determine the sample size required to achieve a desired power for a given *α*-level (and values for *p*_*T*_ and *p*_*S*_), by iteratively testing different sample sizes until a value is found that satisfies the specified design criteria.

To conduct the hypothesis test, a value for *p*_*S*_ (an animal welfare standard) must be specified. This value could be defined as part of procedural documents for that activity, and could involve regulatory and ethical considerations [26]. For example, it could be required that the frequency of mortality in a livestock transport operation be ≤2% [27], that is *p*_*S*_ = 0.02. Alternatively, *p*_*S*_ could be defined relative to the assumed value for the true probability of an adverse outcome (similar to defining the upper limit of the one-sided confidence interval above). For example, it could be required that if the mortality probability is thought to be 0.02 (*p*_*T*_), then there needs to be evidence that the probability is no greater than 50% above this value, that is, *p*_*S*_ = *p*_*T*_(1 + *τ*) = 0.02(1 + 0.5) = 0.03, where *τ* is the desired scaling factor to define the specified hypothesised value.

### Hypothesis testing for published animal welfare studies

Sample sizes for the 10 studies ranged from 11–194,216 (Table 3). We imposed a pass/fail threshold standard for the frequency of the adverse event in question, as proposed by the author of the study or as applied in similar studies (e.g., a non-immediate insensibility threshold standard of 0.05 for captive bolt studies) [22]. We applied a hypothesis-testing approach using an exact test for a binomial proportion.

**Table 3:** The frequency of given adverse events (*p*_*ADV*_) as assessed in 10 published studies of operational animal use activities, and the *p*-values from an exact hypothesis–testing procedure using the alternative hypothesis *H*_*A*_: *p*_*ADV*_ < *p*_*S*_.

Animal species	Operational activity	Adverse event type	Source	*x*_*ADV*_	*n*_*ADV*_	*p*_*ADV*_	*p*_*S*_	*p*-value
Domestic cattle (*Bos taurus*)	Ship transport	Mortality	[6]	742	194,216	0.0038	0.01	0.000
Domestic sheep (*Ovis aries*)	Ship transport	Mortality	[48]	167	9,540	0.018	0.02	0.042
Moose (*Alces alces*)	Helicopter darting	Mortality	[49]	20	2,816	0.0071	0.02	0.000
Domestic cattle (*Bos taurus*)	Captive bolt stunning	Non-immediate insensibility	[21]	31	304	0.10	0.05	1.000
Gray wolf (*Canis lupus*)	Helicopter darting	Mortality	[49]	3	89	0.034	0.02	0.896
Western grey kangaroo (*Macropus fuliginosus*)	Captive bolt euthanasia	Non-immediate insensibility	[30]	1	28	0.036	0.05	0.588
Eastern grey kangaroo (*Macropus giganteus*)	Captive bolt euthanasia	Non-immediate insensibility	[50]	8	21	0.38	0.05	1.000
Brushtail possum (*Trichosurus vulpecula*)	Kill trapping	Sensible after 3 minutes	[51]	1	19	0.053	0.2	0.083
Brushtail possum	Kill trapping	Sensible after 3 minutes	[51]	1	15	0.067	0.2	0.167
Brushtail possum	Kill trapping	Sensible after 3 minutes	[51]	4	11	0.36	0.2	0.950

The number of animals assessed as experiencing adverse events (*x*_*ADV*_) and the total number sampled (*n*_*ADV*_) in each study, are given.

## Results and discussion

### Sample size calculation for estimation of frequency

Tables 4 and 5 indicate the sample sizes required for the case in which the true probability of an adverse outcome is 0.05 and 0.01, respectively.

**Table 4:** Sample size required in order to obtain an expected upper limit of a one-sided exact confidence interval estimate equal to the given values, for a range of confidence levels, assuming the true probability of an adverse outcome = 0.05 (i.e., 5%).

*δ*	Upper limit	Level of confidence			
		80%	90%	95%	99%
0.05	5.25%	6,054	13,381	21,716	42,930
0.10	5.50%	1,674	3,563	5,711	11,174
0.15	5.75%	812	1,678	2,661	5,159
0.20	6.00%	494	996	1,564	3,009
0.25	6.25%	340	670	1,044	1,993
0.30	6.50%	252	488	754	1,430
0.35	6.75%	197	374	575	1,084
0.40	7.00%	159	299	456	855
0.45	7.25%	132	246	373	696
0.50	7.50%	113	207	312	579

The expected proportional distance to the upper limit (*δ*) and the corresponding expected upper limit are given.

**Table 5:** Sample size required in order to obtain an expected upper limit of a one-sided exact confidence interval estimate equal to the given values, for a range of confidence levels, assuming the true probability of an adverse outcome = 0.01 (i.e., 1%).

*δ*	Upper limit	Level of confidence			
		80%	90%	95%	99%
0.05	1.05%	31,528	69,755	113,244	223,931
0.10	1.10%	8,710	18,582	29,799	58,339
0.15	1.15%	4,226	8,754	13,891	26,957
0.20	1.20%	2,570	5,196	8,171	15,732
0.25	1.25%	1,766	3,496	5,454	10,427
0.30	1.30%	1,308	2,544	3,941	7,487
0.35	1.35%	1,020	1,953	3,006	5,678
0.40	1.40%	825	1,559	2,385	4,482
0.45	1.45%	687	1,281	1,950	3,647
0.50	1.50%	584	1,077	1,632	3,038

**Fig 2:**
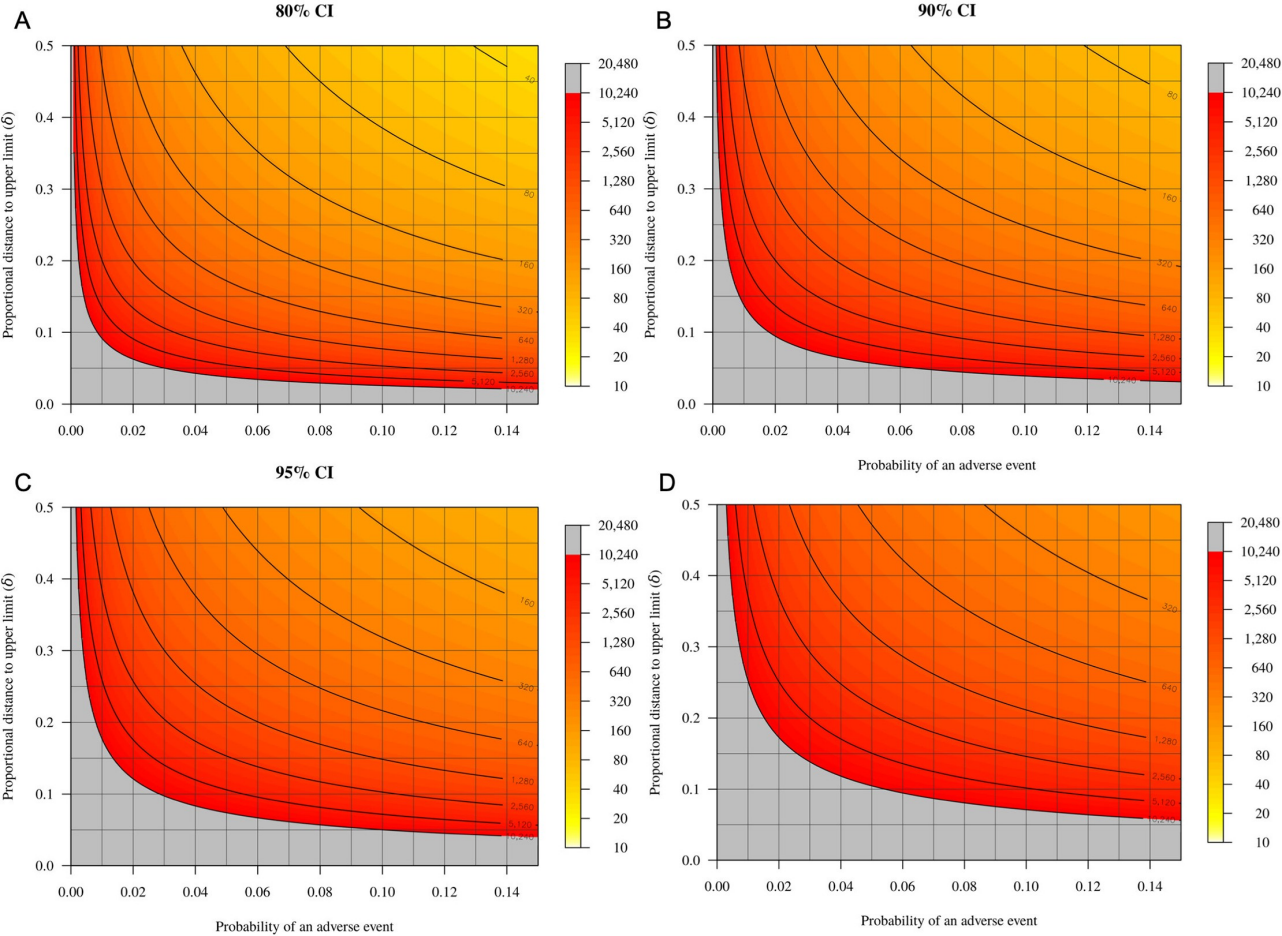
Heat maps illustrating the relationship between the probability of an adverse event (*x*-axis), proportional distance to desired upper limit (*δ*; *y*-axis) and required sample size (yellow-grey gradient) for different required confidence levels (80–99%; a–d).

The expected proportional distance to the upper limit (*δ*, as in Fig 2) and the corresponding expected upper limit are given.

Panels a–d in Fig 2 represent the required sample size for different combinations of the proportional distance to the upper limit and the anticipated probability for exact one-sided 80%, 90%, 95% and 99% confidence interval estimates. The sample size is given up to a maximum of 10,240; larger sample sizes are required for the grey-coloured zone. The required sample size increases in order to obtain a more precise confidence interval estimate (i.e., as *δ* decreases), and also when the probability of an adverse outcome decreases. The sample size also increases when a higher level of confidence for the confidence interval estimate is specified (Fig 2D).

For a given probability of an adverse event, larger sample sizes are required at smaller proportional distances and higher confidence levels. Large samples sizes are required at very low probabilities (<0.02 or 2%). Grey shading indicates sample sizes >10,240.

Our calculations can be used to determine the number of animals that need to be sampled in order to estimate the proportion of adverse animal events with a desired level of precision. An online calculator is provided to enable readers to do this (S2 Appendix).

### Assessment of confidence intervals in published animal welfare studies

**Fig 3:**
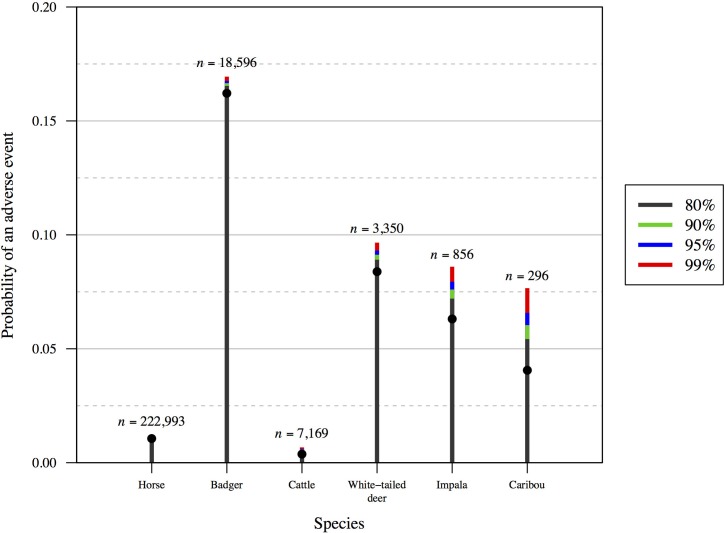
One-sided confidence interval estimates for adverse event frequency estimates from six published animal welfare studies of operational (not research) activities. Point estimates are shown (grey dots), as are the following confidence intervals: 80% (grey), 90% (green), 95% (blue) and 99% (red). For those studies that used sample sizes <1000, the confidence intervals are relatively wide for estimating frequencies, whereas for those that used sample sizes >1000 the confidence intervals are relatively narrow. For study details, see Table 1.

**Fig 4:**
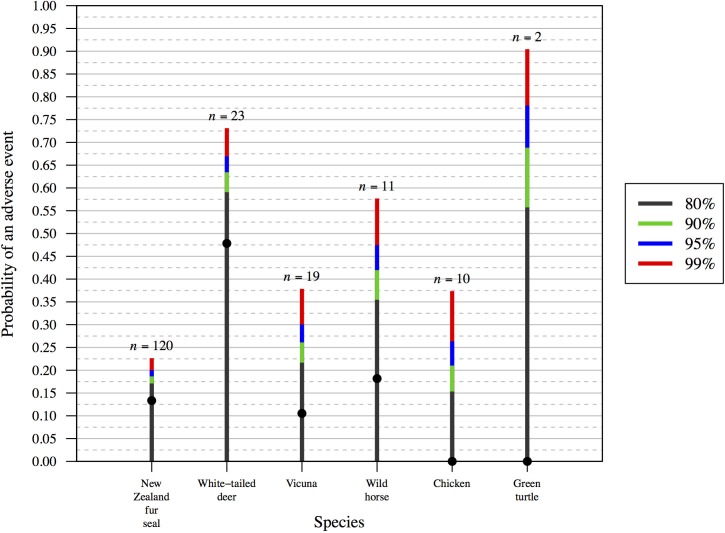
One-sided confidence interval estimates for adverse animal welfare event frequency estimates from six published research trials. Point estimates are shown (grey dots), as are the following confidence intervals: 80% (grey), 90% (green), 95% (blue) and 99% (red). It is evident that for studies that used small sample sizes, the confidence intervals are wide. For study details, see Table 2.

Details for published studies selected are shown in Table 1 (operational activities) and Table 2 (research trials). Confidence intervals for the frequency of adverse animal welfare events are shown in Fig 3 (operational activities) and Fig 4 (research trials). It is evident that the confidence intervals for research trials that used relatively small sample sizes (<100; Fig 4) were much larger when compared with those obtained from monitoring of operational activities that used larger sample sizes (>1000; Fig 3). Indeed, with small sample sizes in research trials, confidence limit intervals can be larger than point estimates of frequency when they are in the range of 0.1–0.2 (Fig 4). The smallest sample sizes in Fig 4 (*n* = 2 and *n* = 10) demonstrate that, even if adverse events are not observed, their probability of occurring could still be considerable when the sample size is small.

More generally, our results have two important implications. First, most animal welfare studies reporting frequency data have not used sample sizes large enough to achieve desirable precision. For example, the study of Hampton and Forsyth [52] attempted to quantify the frequency of non-immediate insensibility in kangaroo shooting and reported a frequency of 0.02 (i.e., 2%) from a sample of 134 animals. According to our results, the minimum sample size should have been ~640 to achieve the minimum levels of precision we considered necessary. The second most important finding of this analysis is that many more animals need to be sampled if the frequency of the adverse event is low (~1%), as is typical in long-running operational activities for which procedures have previously been refined, when compared with more recently developed operational activities, for which the frequency of adverse events is likely to be much higher (often >10%) [28, 53]

## Hypothesis tests

Tables 6 and 7 show the sample sizes required for a range of Type I error rates when the true probability of an adverse outcome is 0.05 and 0.01, respectively.

**Table 6:** Sample size required in order to obtain 80% power to detect that the probability of an adverse outcome is less than *p*_*S*_, for a range of Type I error rates (*α*), assuming that the true probability of an adverse outcome = 0.05 (i.e., 5%).

*τ*	*p*_*S*_	Type I error rate (α)			
		20%	10%	5%	1%
0.05	5.25%	>10,240	>10,240	>10,240	>10,240
0.10	5.50%	5,803	9,302	>10,240	>10,240
0.15	5.75%	2,601	4,205	5,708	9,306
0.20	6.00%	1,540	2,441	3,330	5,319
0.25	6.25%	1,009	1,580	2,208	3,508
0.30	6.50%	709	1,140	1,541	2,501
0.35	6.75%	540	905	1,160	1,868
0.40	7.00%	429	670	930	1,450
0.45	7.25%	339	540	728	1,181
0.50	7.50%	299	448	598	1,004

The scaling factor to the specified value (*τ*, as in Fig 3), and the corresponding specified value (*p*_*S*_), are given.

**Table 7:** Sample size required in order to obtain 80% power to detect that the probability of an adverse outcome is less than *p*_*S*_, for a range of Type I error rates (*α*), assuming that the true probability of an adverse outcome = 0.01 (i.e., 1%).

*τ*	*p_s_*	Type I error rate (α)			
		20%	10%	5%	1%
0.05	1.05%	>10,240	>10,240	>10,240	>10,240
0.10	1.10%	>10,240	>10,240	>10,240	>10,240
0.15	1.15%	>10,240	>10,240	>10,240	>10,240
0.20	1.20%	7,804	>10,240	>10,240	>10,240
0.25	1.25%	5,309	8,097	>10,240	>10,240
0.30	1.30%	3,802	5,804	7,990	>10,240
0.35	1.35%	2,791	4,707	5,994	9,804
0.40	1.40%	2,307	3,602	4,911	7,616
0.45	1.45%	2,004	2,792	3,818	6,417
0.50	1.50%	1,428	2,402	3,315	5,212

The scaling factor to the specified value (*τ*, as in Fig 3), and the corresponding specified value (*p*_*S*_), are given.

Tables 6 and 7 indicate the sample sizes required in order for a study to have 80% power to detect that the observed probability is less than *p*_*S*_ for a range of Type I error rates when the true probability of an adverse outcome is 0.05 and 0.01, respectively. Significance is declared if the *p*-value is less than the Type I error rate. An online calculator was developed to allow readers to determine sample sizes for their own hypothesis tests using the Clopper–Pearson method (S2 Appendix).

**Fig 5:**
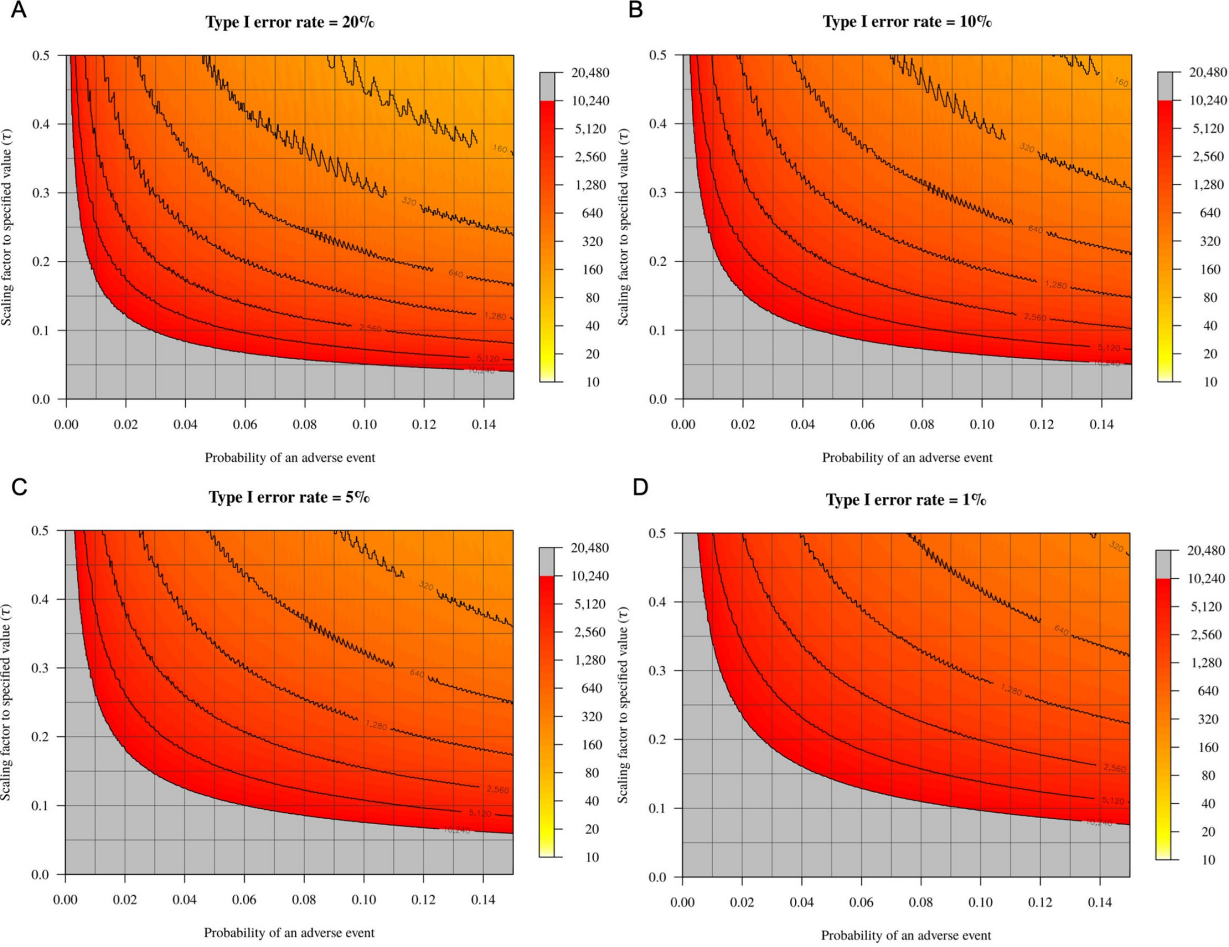
**Heat maps illustrating the relationship between the probability of an adverse event (*x*-axis), the scaling factor to specified value (*y*-axis), and the required sample size (yellow-grey gradient) for different Type I error rates (1–20%; a–d).** For a given probability of an adverse event, larger sample sizes are required with low Type I error rates and at low scaling factors. The sample size is given up to a maximum of 10,240; larger sample sizes are required for the grey-coloured zone.

The plots in Fig 5A–5D represent the sample sizes required in order to estimate, with 80% power for the stated Type I error rate, that the observed probability is less than *p*_*S*_ for a range of Type I error rates. In this instance, the contour lines are not smooth because of the discrete nature of the binomial distribution. There was a similar pattern in the sample size required for hypothesis testing, as was observed for confidence interval estimation (Fig 5). The required sample size increases when the hypothesised value of interest is closer to the true probability of an adverse outcome (i.e., as *τ* decreases), and when the probability of an adverse outcome is smaller. The sample size also increases as the Type I error rate (i.e., the *α*-level, or the *p*-value at which the *H*_*0*_ would be rejected), decreases (Fig 5).

### Assessment of published hypothesis tests

The *p*-values shown in Table 3 reveal that not all studies produced significant results (i.e., they did not pass the threshold) despite some point estimates of adverse event frequency being below the threshold level. For example, in the captive bolt study of Hampton [30], the reported point estimate (0.04) was less than the threshold standard (0.05), but the statistical power was too low to provide a statistically significant comparison with the animal welfare standard (*p*-value 0.588) due to the small sample size (*n* = 28).

Even for relatively well-developed animal welfare fields such as slaughter of livestock, the issue of sample size has received little attention. For example, animal-based data derived from abattoir auditing is often compared with a threshold of 95% effective stunning [22, 54, 55], equivalent to a frequency of non-immediate insensibility of 0.05, requiring the sample sizes shown in Table 6. However, reported sample sizes from such studies often fail to reach the minimum number we calculated for this purpose (299) [21, 30], even with relatively high error rates (20%) and scaling factors (0.5; Table 6). We encourage future animal welfare monitoring studies to heed the lessons demonstrated in the examples in Table 3 and to provide the resources needed in order to sample enough animals so as to produce statistically robust results. Providing adequate resources is likely to be particularly important when there is public scrutiny of an animal welfare assessment, considering the potential cost of a false positive result. In this context, a false positive result would take the form of a sample indicating that adverse frequency exceeded a threshold, when the true frequency in the population did not. In a contentious field, such as livestock transport [5, 8], the public cost of such a false positive result could be considerable.

## Pilot studies and research trials

For previously untested research or animal management techniques, research trials (pilot studies or validation studies) are typically advised or required before approval is given for operational use [9]. The three Rs approach to minimising animal welfare impacts in research indicates that the number of animals affected should be minimised wherever possible [56, 57]. This logically leads to a compromise between statistical rigour (maximising the sample sizes in order to create greater confidence) and animal impacts (minimising the numbers of animals affected) for trials of novel animal manipulation techniques [58]. Hence, small sample sizes must be used according to the precautionary principle, and tolerance levels must be high in order for outcome data to exceed specified thresholds [59]. For this reason, many pilot studies use small sample sizes (e.g., ~10 animals) and often do not report confidence intervals for frequency data [29, 30, 50]. For example, in New Zealand, lethal traps for carnivorous and omnivorous mammals are required to pass animal welfare threshold tests designed from guidelines produced by ISO (the International Organization for Standardization) [58]. These specify that 90% confidence is required that insensibility occurs within 3 minutes in >70% of test animals [59]. Sample sizes used are typically 10–19 animals (Table 3). We suggest that in this context, smaller sample sizes could be used by allowing high tolerance.

Some research trials involve rare wildlife species that are of conservation concern. For such species (e.g., Amur tigers (*Panthera tigris altaica*) [60] and Tasmanian devils (*Sarcophilus harrisii*) [61]), attaining large sample sizes may be impossible. We emphasise that our guidelines in this study are primarily designed for monitoring of operational activities, allowing large sample sizes, rather than research programs or pilot studies of new technologies [9]. In these situations, it would be worth considering using alternative methods for estimating sample size requirements e.g., [34, 36].

### Recommendations for designing welfare monitoring programs

The designers of animal welfare monitoring programs should use the guidelines presented here to ensure that the desired level of precision is achieved. For activities that demonstrate a relatively high frequency of adverse events (e.g., 0.12 [12%] non-fatal wounding for amateur shooting of European rabbits (*Oryctolagus cuniculus*)) [16], relatively low sample sizes can be used. However, for techniques that achieve a relatively low frequency of adverse events (e.g., 0.01 or 1% mortalities for helicopter darting of moose (*Alces alces*) [49], or pole syringe capture of kangaroos [62]), much higher sample sizes must be used.

## Conclusions

The sample sizes used to estimate the frequency of adverse events in animal welfare studies have been high variable. The desired level of precision for the outcome(s) of interest should be reported in all publications, along with the required sample size(s). The guidelines presented here should be used to determine the number of animals to be sampled in order to estimate the proportion of adverse animal events with a desired level of precision.

## Supporting information

S1 Appendix (DOCX)

S2 Appendix (DOCX)

## References

[1] Dubois S, Fenwick N, Ryan EA, Baker L, Baker SE, Beausoleil NJ, et al International consensus principles for ethical wildlife control. Conservation Biology. 2017;31:753–60. 10.1111/cobi.12896 28092422

[2] Dawkins MS. A user's guide to animal welfare science. Trends in Ecology & Evolution. 2006;21(2):77–82. 16701478 10.1016/j.tree.2005.10.017

[3] Millman ST, Johnson AK, O'Connor AM, Zanella AJ. Animal welfare and epidemiology—across species, across disciplines, and across borders. Journal of Applied Animal Welfare Science. 2009;12(2):83–7. 10.1080/10888700902719542 19319710

[4] Hampton J. Animal welfare for wild herbivore management. Perth, Australia: Murdoch University; 2017.

[5] Caulfield MP, Cambridge H, Foster SF, McGreevy PD. Heat stress: A major contributor to poor animal welfare associated with long-haul live export voyages. The Veterinary Journal. 2014;199(2):223–8. 10.1016/j.tvjl.2013.09.018 24157340

[6] Perkins N, O’Hara M, Creeper J, Moore J, Madin B, McCarthy M. Identifying the causes of mortality in cattle exported to the Middle East. North Sydney, Australia: Meat & Livestock Australia, 2015.

[7] Schipp M. The welfare of livestock transported by sea: Australia’s experience. The Veterinary Journal. 2013;3(196):282–3. 10.1016/j.tvjl.2013.04.004 23732076

[8] Foster SF, Overall KL. The welfare of Australian livestock transported by sea. The Veterinary Journal. 2014;200(2):205–9. 10.1016/j.tvjl.2014.03.016 24742871

[9] Hampton JO, Hyndman TH, Laurence M, Perry AL, Adams P, Collins T. Animal welfare and the use of procedural documents: limitations and refinement. Wildlife Research. 2016;43(7):599–603.

[10] Sharp T, Saunders G. A model for assessing the relative humaneness of pest animal control methods: Department of Agriculture, Fisheries and Forestry; 2011.

[11] Descovich K, McDonald I, Tribe A, Phillips C. A welfare assessment of methods used for harvesting, hunting and population control of kangaroos and wallabies. Animal Welfare. 2015;24(3):255–65.

[12] Commonwealth of Australia. National code of practice for the humane shooting of kangaroos and wallabies for non-commercial purposes. Canberra, Australian Capital Territory, Australia: Department of Environment and Heritage, 2008.

[13] Commonwealth of Australia. National code of practice for the humane shooting of kangaroos and wallabies for commercial purposes. Canberra, Australian Capital Territory, Australia: Department of Environment and Heritage, 2008.

[14] McMahon CR, Hindell MA, Harcourt RG. Publish or perish: why it’s important to publicise how, and if, research activities affect animals. Wildlife Research. 2012;39(5):375–7.

[15] Williams R, Harkins L, Hammond C, Wood J. Racehorse injuries, clinical problems and fatalities recorded on British racecourses from flat racing and National Hunt racing during 1996, 1997 and 1998. Equine veterinary journal. 2001;33(5):478–86. 11558743 10.2746/042516401776254808

[16] Hampton J, Forsyth D, Mackenzie D, Stuart I. A simple quantitative method for assessing animal welfare outcomes in terrestrial wildlife shooting: the European rabbit as a case study. Animal Welfare. 2015;24(3):307–17.

[17] McKenzie J, Page B, Goldsworthy SD, Hindell MA. Behavioral responses of New Zealand fur seals (*Arctophoca australis forsteri*) to darting and the effectiveness of midazolam and tiletamine‐zolazepam for remote chemical immobilization. Marine Mammal Science. 2013;29(2):241–60.

[18] Parfit D. Equality and priority. Ratio. 1997;10(3):202–21.

[19] Lassen J, Sandøe P, Forkman B. Happy pigs are dirty!–conflicting perspectives on animal welfare. Livestock Science. 2006;103(3):221–30.

[20] Limon G, Guitian J, Gregory NG. An evaluation of the humaneness of puntilla in cattle. Meat Science. 2010;84(3):352–5. 10.1016/j.meatsci.2009.09.001 20374796

[21] Doyle R, Coleman G, McGill D, Reed M, Ramdani W, Hemsworth P. Investigating the welfare, management and human-animal interactions of cattle in four Indonesian abattoirs. Animal Welfare. 2016;25(2):191–7.

[22] Grandin T. Maintenance of good animal welfare standards in beef slaughter plants by use of auditing programs. Journal of the American Veterinary Medical Association. 2005;226(3):370–3. 15702685 10.2460/javma.2005.226.370

[23] Hunt JR, Dean RS, Davis GN, Murrell JC. An analysis of the relative frequencies of reported adverse events associated with NSAID administration in dogs and cats in the United Kingdom. The Veterinary Journal. 2015;206(2):183–90. 10.1016/j.tvjl.2015.07.025 26361747

[24] McMillan M, Darcy H. Adverse event surveillance in small animal anaesthesia: an intervention-based, voluntary reporting audit. Veterinary Anaesthesia and Analgesia. 2016;43(2):128–35. 10.1111/vaa.12309 26479166

[25] Hampton J, Finch N, Watter K, Amos M, Pople T, Moriarty A, et al A review of methods used for the capture of wild deer in Australia Australian Mammalogy. 2018; Online Early.

[26] Mellor DJ, Bayvel ACD. New Zealand's inclusive science-based system for setting animal welfare standards. Applied Animal Behaviour Science. 2008;113(4):313–29.

[27] The Australian Standards for the Export of Livestock. Investigations into mortalities. Canberra, Australia: Australian Govermnet Department of Agriculture and Water Resources, 2016.

[28] Hampton J, Robertson H, Adams P, Hyndman T, Collins T. An animal welfare assessment framework for helicopter darting: a case study with a newly developed method for feral horses. Wildlife Research. 2016;43:429–37.

[29] Kelly D. Report into the Camel Program Witjera National Park: May 2005. Adelaide, Australia: South Australian Department for Environment and Heritage, 2005.

[30] Hampton JO. Gunpowder-powered captive bolts for the euthanasia of kangaroo pouch young. Australian Mammalogy. 2018;Online Early.

[31] Newcombe RG. Two‐sided confidence intervals for the single proportion: comparison of seven methods. Statistics in Medicine. 1998;17(8):857–72. 9595616 10.1002/(sici)1097-0258(19980430)17:8<857::aid-sim777>3.0.co;2-e

[32] Gardner MJ, Altman DG. Confidence intervals rather than P values: estimation rather than hypothesis testing. British Medical Journal. 1986;292(6522):746–50. 3082422 10.1136/bmj.292.6522.746 PMC1339793

[33] Naing L, Winn T, Rusli B. Practical issues in calculating the sample size for prevalence studies. Archives of Orofacial Sciences. 2006;1:9–14.

[34] Clopper CJ, Pearson ES. The use of confidence or fiducial limits illustrated in the case of the binomial. Biometrika. 1934;26(4):404–13.

[35] Wilson EB. Probable inference, the law of succession, and statistical inference. Journal of the American Statistical Association. 1927;22(158):209–12.

[36] Prendergast LA, Staudte RG. Better than you think: interval estimators of the difference of binomial proportions. Journal of Statistical Planning and Inference. 2014;148:38–48.

[37] Thulin M. The cost of using exact confidence intervals for a binomial proportion. Electronic Journal of Statistics. 2014;8(1):817–40.

[38] Byrne AW, O’Keeffe J, Fogarty U, Rooney P, Martin SW. Monitoring trap-related injury status during large-scale wildlife management programmes: an adaptive management approach. European Journal of Wildlife Research. 2015;61(3):445–55.

[39] Stafford K, Mellor D, Todd S, Gregory N, Bruce R, Ward R. The physical state and plasma biochemical profile of young calves on arrival at a slaughter plant. New Zealand Veterinary Journal. 2001;49(4):142–9. 10.1080/00480169.2001.36222 16032182

[40] Webb SL, Lewis JS, Hewitt DG, Hellickson MW, Bryant FC. Assessing the helicopter and net gun as a capture technique for white-tailed deer. Journal of Wildlife Management. 2008;72(1):310–4.

[41] Lewis A, Pinchin A, Kestin S. Welfare implications of the night shooting of wild impala (*Aepyceros melampus*). Animal Welfare. 1997;6(2):123–31.

[42] Valkenburg P, Tobey RW, Kirk D. Velocity of tranquilizer darts and capture mortality of caribou calves. Wildlife Society Bulletin. 1999;27(4):894–6.

[43] Kilpatrick HJ, DeNicola AJ, Ellingwood MR. Comparison of standard and transmitter-equipped darts for capturing white-tailed deer. Wildlife Society Bulletin. 1996;24:306–10.

[44] Bonacic C, Macdonald D. The physiological impact of wool-harvesting procedures in vicunas (*Vicugna vicugna*). Animal Welfare. 2003;12(3):387–402.

[45] Raj A, O'Callaghan M. Evaluation of a pneumatically operated captive bolt for stunning/killing broiler chickens. British Poultry Science. 2001;42(3):295–9. 10.1080/00071660120055232 11469546

[46] Flint M, Mills PC, Loban F, Simpson T, Lui S, Fujii R, et al Development of a humane slaughter device for green turtles for use by traditional owners in the Torres Strait Islands, Australia. PloS One. 2017;12(1):e0167849 10.1371/journal.pone.0167849 28076432 PMC5226787

[47] Röhmel J, Mansmann U. Unconditional non‐asymptotic one‐sided tests for independent binomial proportions when the interest lies in showing non‐inferiority and/or superiority. Biometrical Journal: Journal of Mathematical Methods in Biosciences. 1999;41(2):149–70.

[48] Richards R, Norris R, Dunlop R, McQuade N. Causes of death in sheep exported live by sea. Australian Veterinary Journal. 1989;66(2):33–8. 2712765 10.1111/j.1751-0813.1989.tb03011.x

[49] Arnemo JM, Ahlqvist P, Andersen R, Berntsen F, Ericsson G, Odden J, et al Risk of capture-related mortality in large free-ranging mammals: experiences from Scandinavia. Wildlife Biology. 2006;12(1):109–13.

[50] Sharp TM, McLeod SR, Leggett KEA, Gibson TJ. Evaluation of a spring-powered captive bolt gun for killing kangaroo pouch young. Wildlife Research. 2015;41(7):623–32.

[51] Warburton B, Hall JV. Impact momentum and clamping force thresholds for developing standards for possum kill traps. New Zealand Journal of Zoology. 1995;22:39–44.

[52] Hampton JO, Forsyth DM. An assessment of animal welfare for the culling of peri-urban kangaroos. Wildlife Research. 2016;43(3):261–6.

[53] Hampton J, Skroblin A, De Ridder TR, Perry AL. Chemical immobilisation and rangeland species: assessment of a helicopter darting method for Australian cattle. The Rangeland Journal. 2016;38:533–40.

[54] Grandin T. Effect of animal welfare audits of slaughter plants by a major fast food company on cattle handling and stunning practices. Journal of the American Veterinary Medical Association. 2000;216(6):848–51. 22570895 10.2460/javma.2000.216.848

[55] Grandin T. Progress and challenges in animal handling and slaughter in the US. Applied Animal Behaviour Science. 2006;100(1):129–39.

[56] Balls M, Goldberg AM, Fentem JH, Broadhead CL, Burch RL, Festing MF, et al The three Rs: the way forward. The report and recommendations of ECVAM workshop 11. Alternatives to Laboratory Animals. 1995;23:838–66. 11660368

[57] Hooijmans CR, Leenaars M, Ritskes-Hoitinga M. A gold standard publication checklist to improve the quality of animal studies, to fully integrate the Three Rs, and to make systematic reviews more feasible. Alternatives to Laboratory Animals. 2010;38:167–82. 20507187 10.1177/026119291003800208

[58] International Organisation for Standardisation. Animal (mammal) traps–part 4: methods for testing killing-trap systems used on land or underwater. Geneva, Switzerland: International Organisation for Standardisation, 1999.

[59] Morriss GA, Warburton B. Modifying the Victor^®^ Easy Set^®^ rat trap to improve the animal welfare of stoats and ship rats trapped in New Zealand. PloS One. 2014;9(2):e86760 10.1371/journal.pone.0086760 24505264 PMC3914800

[60] Goodrich JM, Kerley LL, Schleyer BO, Miquelle DG, Quigley KS, Smirnov YN, et al Capture and chemical anesthesia of Amur (Siberian) tigers. Wildlife Society Bulletin. 2001;29:533–42.

[61] Jones M, Hamede R, McCallum H. The Devil is in the detail: conservation biology, animal philosophies and the role of animal ethics committees In: Banks P, Lunney D, Dickman C, editors. Science under siege: zoology under threat. Sydney, Australia: Royal Zoological Society of New South Wales; 2012 p. 79–88.

[62] King W, Wilson M, Allen T, Festa-Bianchet M, Coulson G. A capture technique for free-ranging eastern grey kangaroos (*Macropus giganteus*) habituated to humans. Australian Mammalogy. 2011;33(1):47−51.

