# Supplementary material for: How many to sample? Statistical guidelines for monitoring animal welfare outcomes
Source: PLoS One. 2019 Jan 30;14(1):e0211417. doi: 10.1371/journal.pone.0211417 (PMC6353194; doi:10.1371/journal.pone.0211417)
Supplement: S1 Appendix — (DOCX) [file pone.0211417.s001.docx]

**S1 Appendix. Comparison of the Clopper–Pearson ‘exact’ method and the Wilson method for estimating one-tailed confidence intervals and hypothesis tests for binomial proportions.**

It is well known that the actual coverage of the Clopper–Pearson (1934) ‘exact’ confidence intervals (CIs) for *p* can be larger than the nominal confidence level, unless *n* is large, and hence this method is considered ‘conservative’ [1]. The Wilson method [2], also called the ‘score CI’, is one of many alternative methods considered to have coverage closer to the nominal confidence level (e.g., [1]). We conducted five comparisons of the performance of the Clopper–Pearson exact method and the Wilson method, when applied to the question of how many animals need to be monitored to quantify the frequency of adverse welfare events. All but one of these comparisons (the first simulation) involve applying the Wilson method to the same data and simulations as reported for the Clopper–Pearson exact method (hereinafter the ‘exact method’) in the main text.

We first conducted simulations to compare the coverage, relative width and the expected upper limit of one-sided 95% CIs estimated by the exact method and the Wilson method. The simulations were performed for a range of values of *p* between 0.01 and 0.15 and for sample sizes (*n*) between 10 and 10,000. The results for only a subset of the scenarios are presented here, but they are indicative of the full results, and are summarized in the 12 panels in Fig S1. For each scenario 5000 sets of data were simulated, and for each simulated dataset 95% one-sided CIs were calculated. The width of the CI, and whether or not the true value included within the interval, were determined and stored.

**
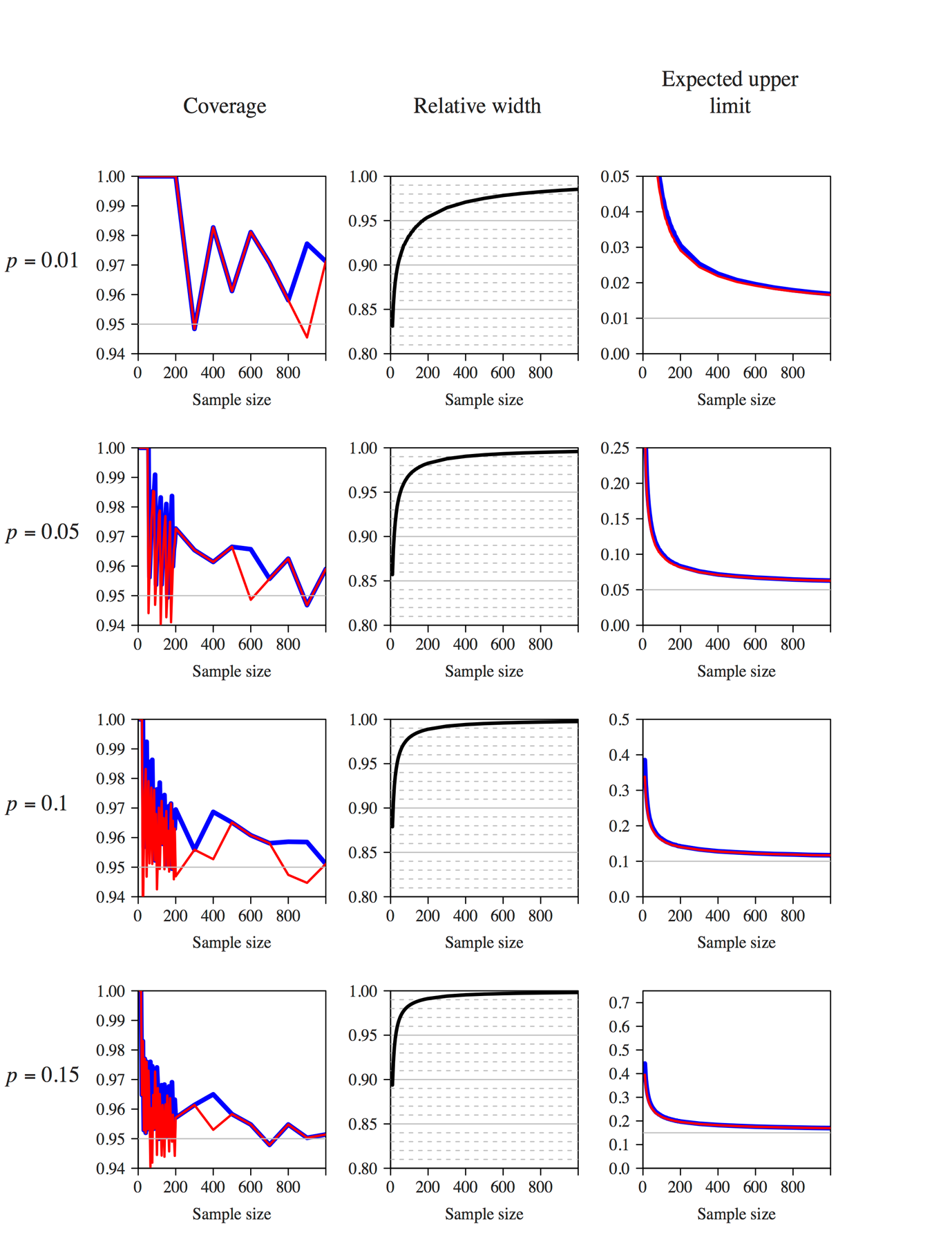
**

**Fig S1. Results of simulations conducted to compare (i) coverage (left column), (ii) relative width (centre column) and (iii) expected upper limit (right column) of the exact method and the Wilson method for four *p* values (from top to bottom rows: 0.05, 0.01, 0.10 and 0.15) .** Coverage (left column) for an estimator should ideally always exceed the nominal 95% value indicated by the grey line. For relative width (centre column), a value <1 indicates that the Wilson confidence interval is narrower than the exact confidence interval. The expected value of the upper limit (right column) provides context for the relative width figures, with the grey line indicating the true value: for example, the relative width figures suggest that the Wilson method is much better when sample sizes are small, but in those situations the upper limit is often very large relative to the true probability value.

Fig S1 illustrates two important differences between the exact and Wilson methods. First, whereas the nominal coverage was always achieved by the exact method (i.e., the true value for *p* was within at least 95% of the confidence intervals calculated from the simulated datasets), it was frequently not achieved by the Wilson method at sample sizes of less than 200 and when *p* was ≥0.05 (Fig S1). Second, the relative widths of the one-sided CIs estimated by the Wilson method were always narrower than those estimated by the exact method, although the difference was attenuated with increasing sample size. However, although the relative widths of the CIs estimated with the Wilson method were much narrower at sample sizes of less than ~100, particularly as *p* became very small, in these situations the upper limit calculated by either method was many times (i.e., >4 times) greater than the true value of *p*, making reliable inference about *p* difficult. Furthermore, the coverage for both methods tended to 1.0 in these scenarios. That is, while the Wilson method may be better in some respects, neither method worked well in these cases because a larger sample size is required. With an adequate sample size, there appeared to be little practical difference between the methods.

Second, we evaluated the required sample size for different combinations of the proportional difference to the upper limit and the anticipated probability for one-sided 80%, 90%, 95% and 99% CI estimates using the Wilson method, and compared them with those achieved using the exact method (Fig 2 in the main text). Panels in Fig S2 represent the required sample size for different combinations of the proportional distance to the upper limit and the anticipated probability of an adverse event for the exact method. The sample size is given up to a maximum of 10,240; larger sample sizes are required for the grey-coloured zone. As was found for the exact method (Fig 2 in the main text), the required sample size increased in order to obtain a more precise confidence interval estimate (i.e., as *δ* decreased), and also when the probability of an adverse outcome decreased. In some instances, the sample size required to achieve the desired confidence interval using the Wilson method was less than that required using the exact method (particularly for 80% and 90% CIs), but the differences were not substantial. Whichever method was used, a sample size of at least several hundred was required to obtain a precise estimate when the probability of an adverse outcome was small (Fig 2, Fig S2).


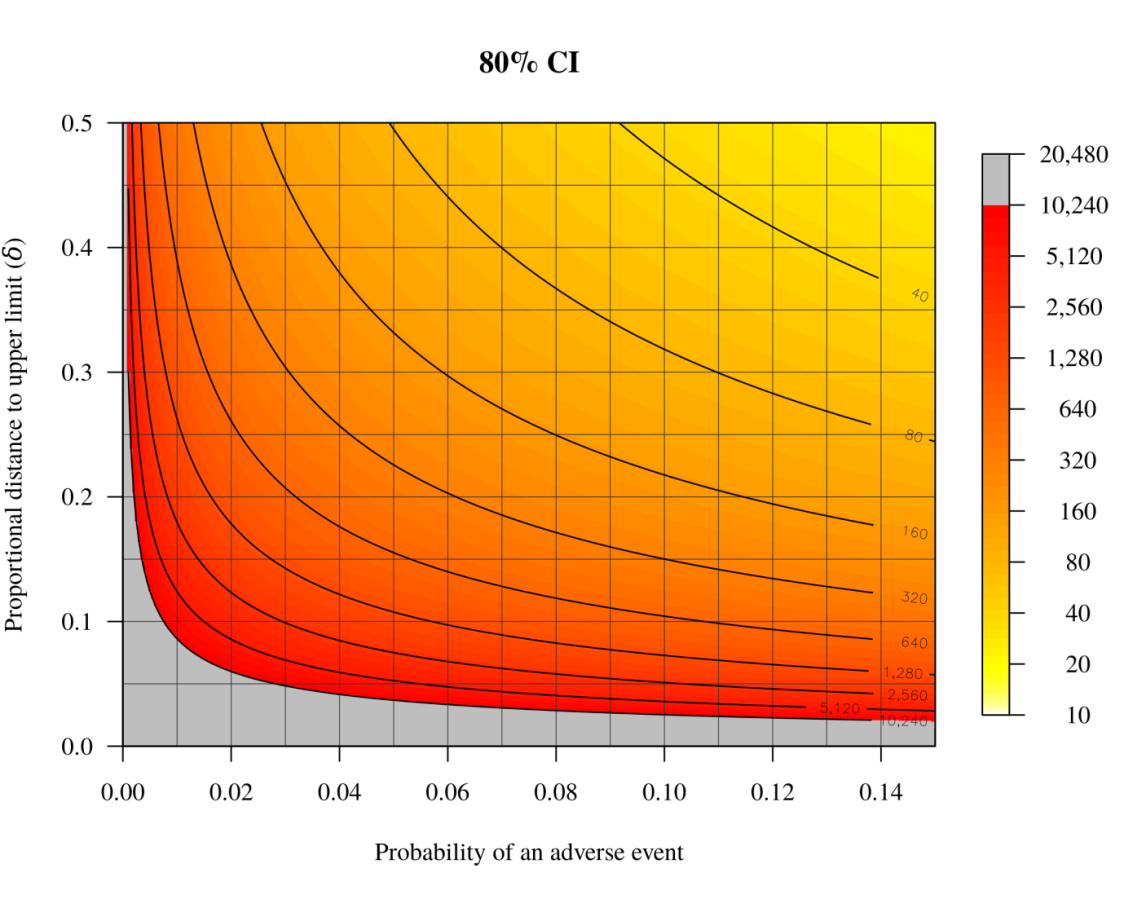

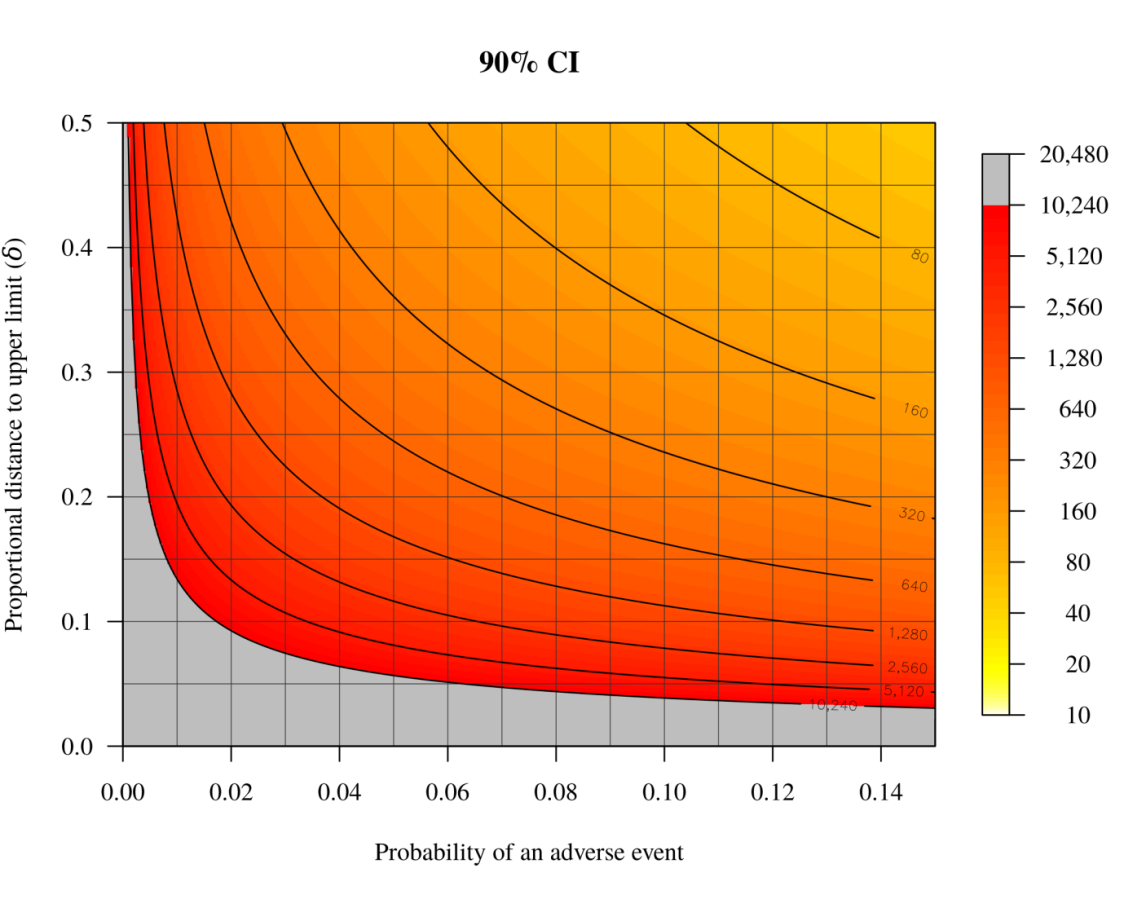

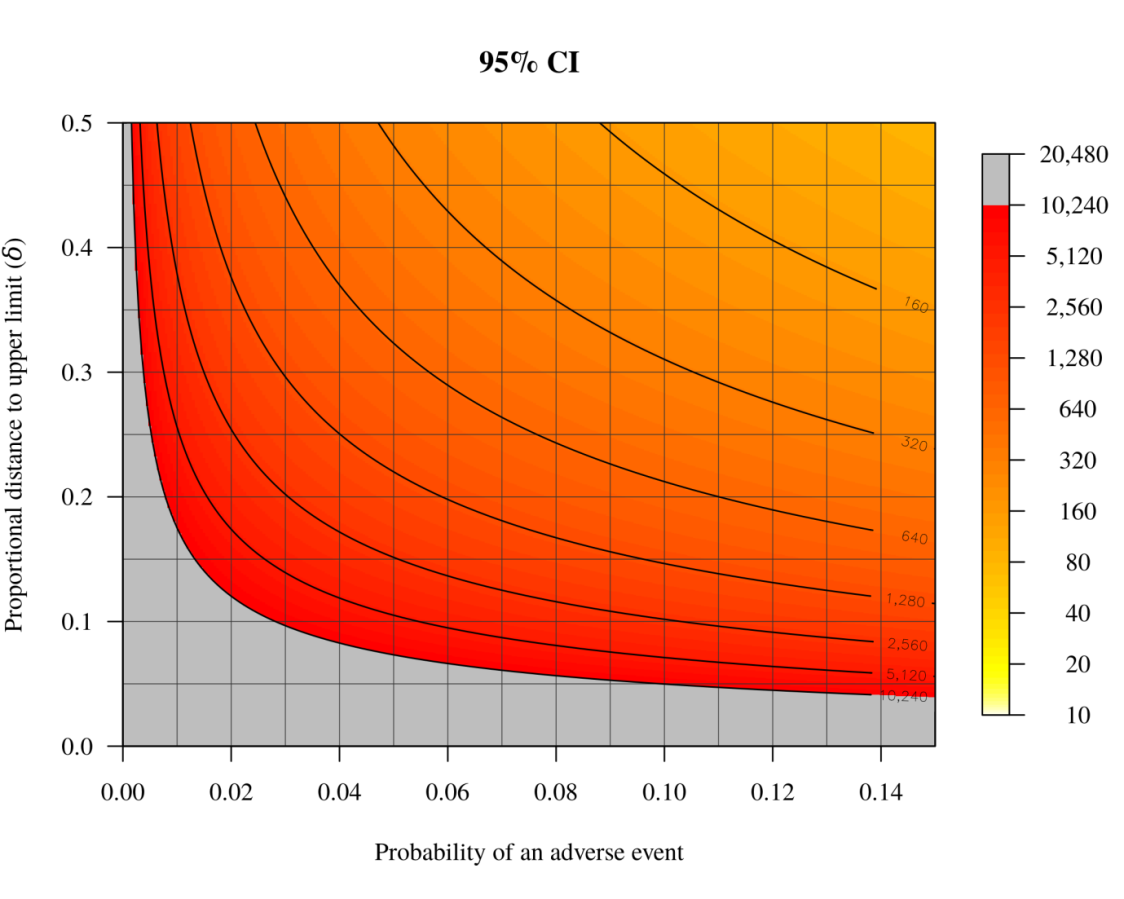

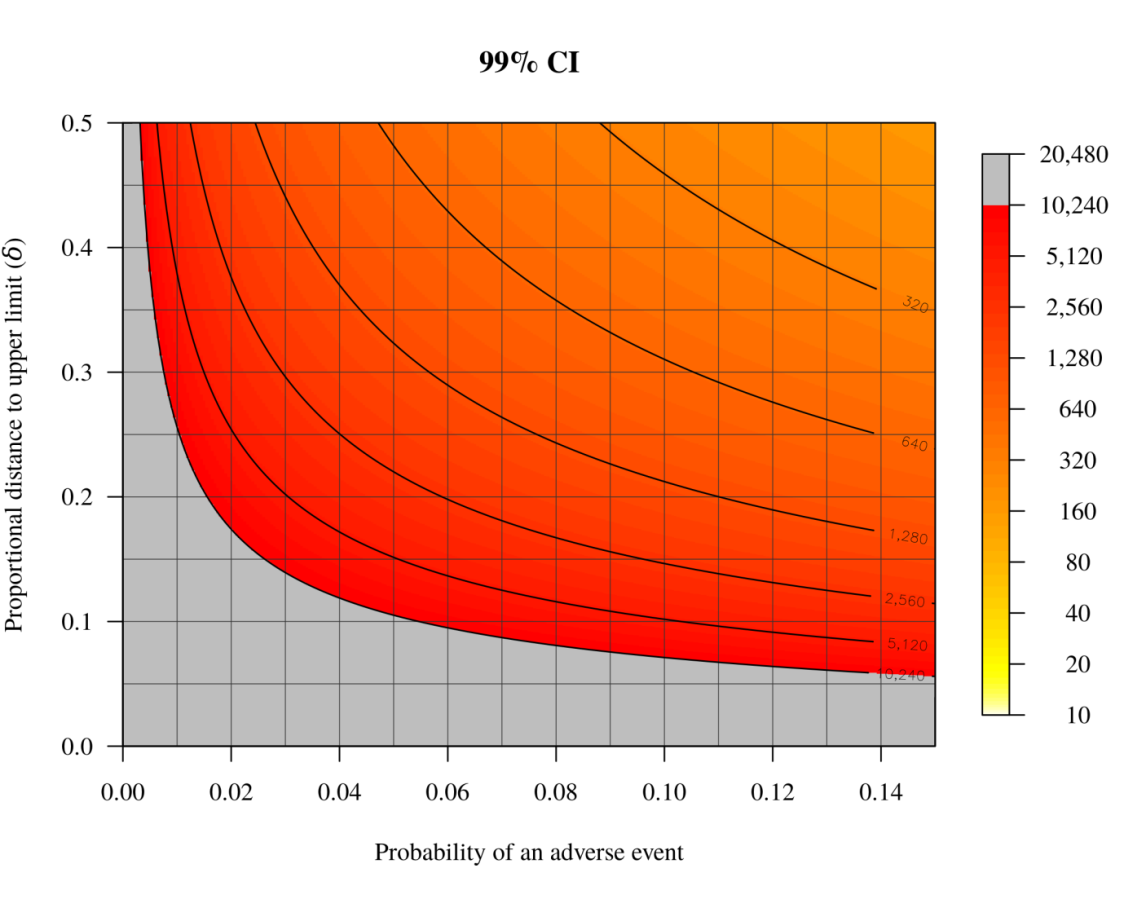


**Fig S2.** **Heat maps illustrating the relationship between the probability of an adverse event (*x*-axis), the proportional distance to the desired upper limit (*δ*; *y*-axis), and the required sample size (yellow-grey gradient) for four specified confidence levels (80%, 90%, 95%, 99%), using the Wilson method.** These heat maps can be contrasted with those estimated using the exact method (Fig 2 in the main text). For a given probability of an adverse event, larger sample sizes were required at smaller proportional distances and higher confidence levels. Large samples sizes were required at very low probabilities (<0.02 or 2%). Grey shading indicates sample sizes of >10,240.

Third, we used the Wilson method to determine the sample sizes required in order to estimate, with 80% power, that the observed probability was less than a specified value of interest (*p_S_*) for four Type I error rates: 1%, 5%, 10% and 20% (Fig S3). Compared with the corresponding sample sizes required when using the exact method (Fig 3), the sample sizes required when using the Wilson method were similar (Fig S3), except that smaller sample sizes were needed when using the Wilson method when the Type I error rate was 20%. Since most practitioners would, by convention, use a Type I error rate of 1% or 5%, the differences observed were unimportant.


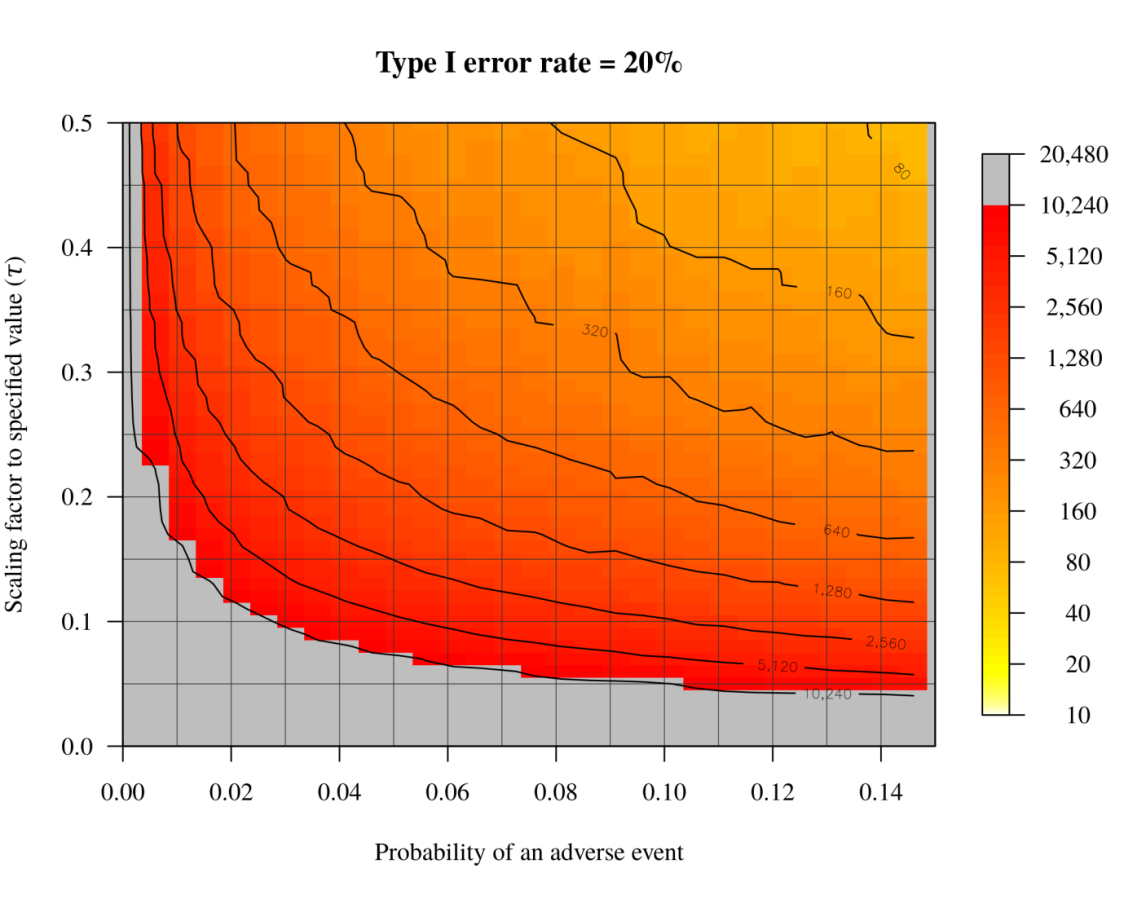

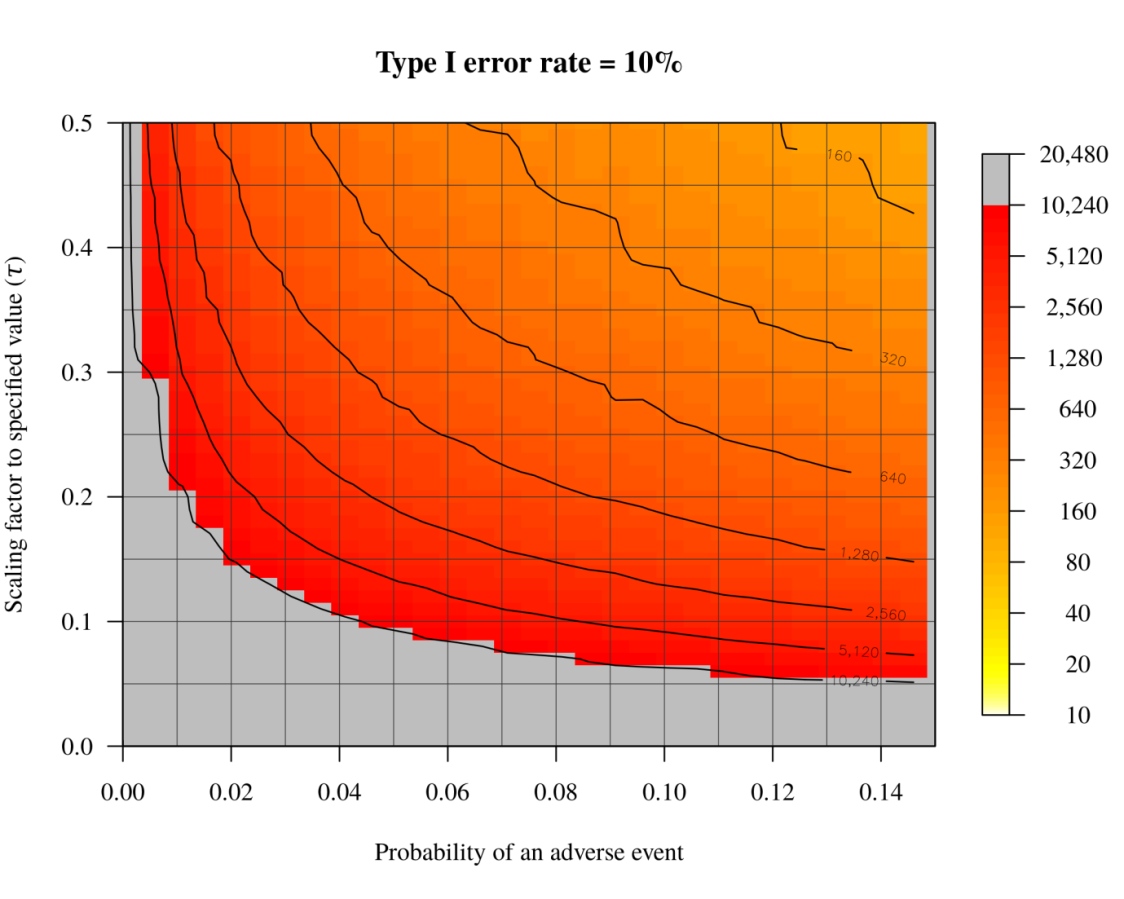

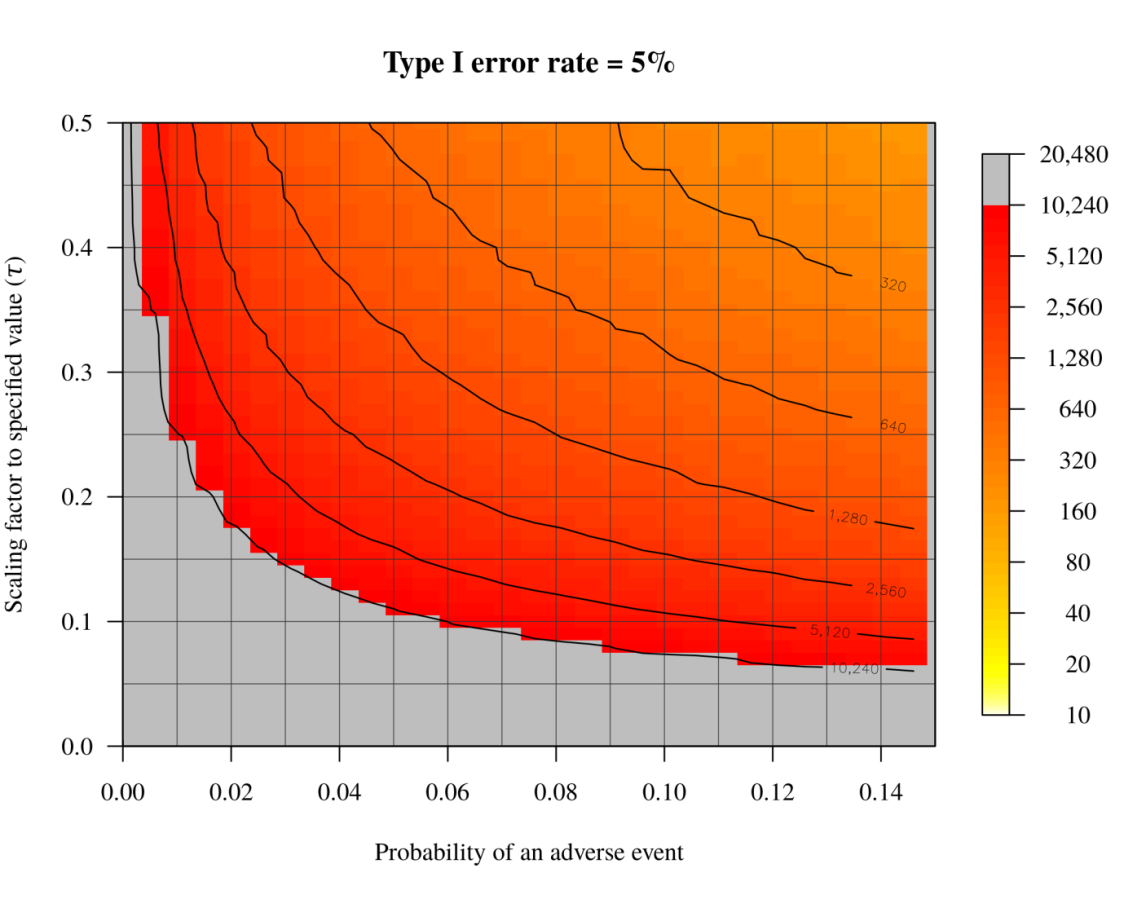

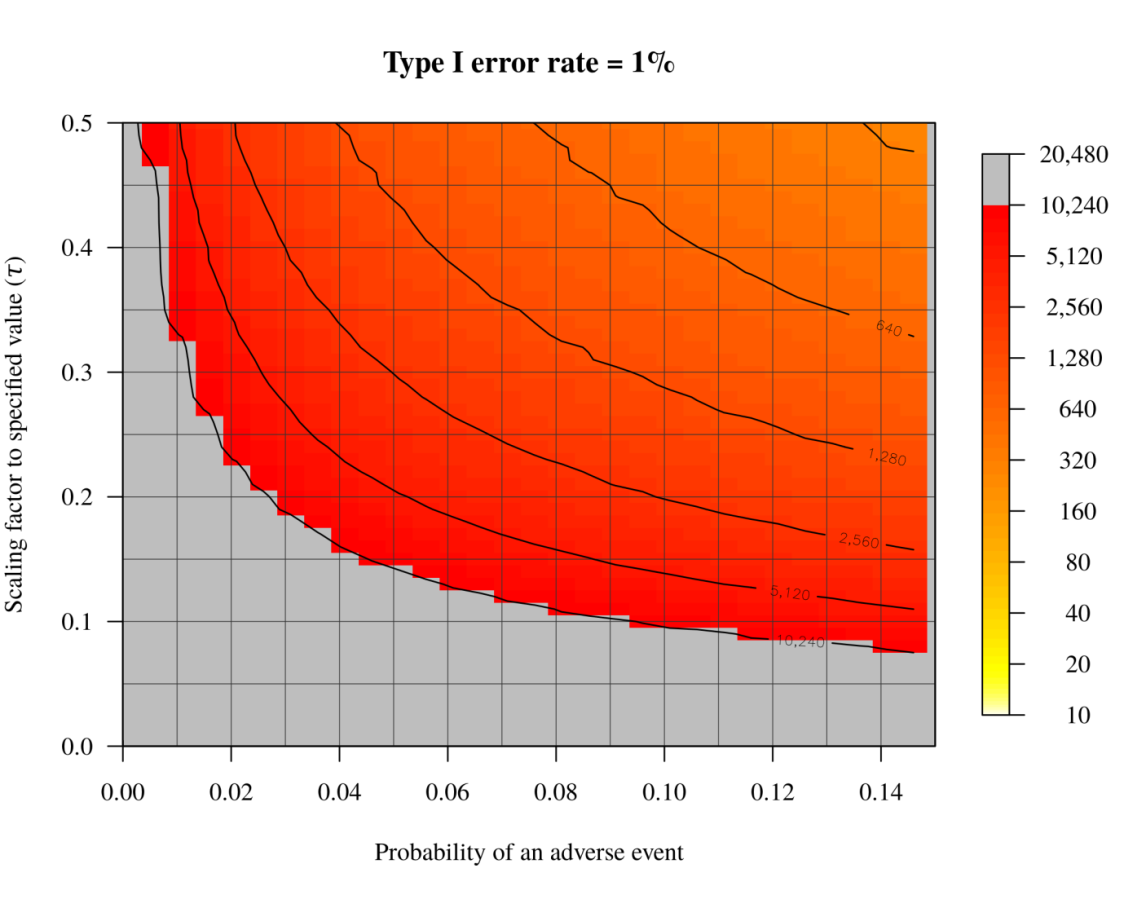


**Fig S3. Heat maps illustrating the relationship between the probability of an adverse event (*x*-axis), the scaling factor to the specified value (*y*-axis), and the required sample size (yellow-grey gradient) for four Type I error rates (1–20).** For a given probability of an adverse event, larger sample sizes were required with low Type I error rates and at low scaling factors. The sample size is given up to a maximum of 10,240; larger sample sizes were required for the grey-coloured zone.

Fourth, we tested the performance of the two methods when used to conduct hypothesis tests about the frequency (probability) of adverse events (*p*_ADV_) observed in the 10 operational animal use activities reported in Table 3 of the main text. This analysis examined two scenarios. In the first scenario, the null hypothesis was that the observed *p*_ADV_ was greater than or equal to the specified value of interest, with the alternative hypothesis being that *p*_ADV_ was less than the specified value of interest (i.e., ‘guilty until proven innocent’). In the second scenario, the null hypothesis was that the observed *p*_ADV_ was less than or equal to the specified value of interest, with the alternative hypothesis being that *p*_ADV_ was greater than the specified value of interest (i.e., ‘innocent until proven guilty’). These analyses were performed in R version 3.4.4 [3], using the prop.test() function in the base stats R package. The results for the two scenarios are shown in Tables S1 and S2. The Wilson method usually gave smaller ­*p*-values than the exact method in both scenarios, but in none of the 10 studies was the difference large enough to change our inferences about the probability of an adverse event. The software gave a warning that the underlying assumptions of the Wilson method may have been violated due to small expected values (resulting from a combination of a small sample size and/or low *p*_ADV_) for the six studies in which the total number sampled (*n_ADV_*) was less than or equal to 89 (Tables S1, S2). This comparison indicated that there was not a great practical difference between the results obtained by the two methods, and that if anything the practitioner could have more confidence in the exact test because its assumptions were less prone to being violated at smaller sample sizes.

**Table S1. A comparison of the probability that the frequency of observed adverse events (p_ADV_) is greater than the threshold (null) value (i.e., ‘guilty until proven innocent’) using the Clopper–Pearson (‘exact’) and Wilson methods.** The source of each study is given in Table 3 in the main text. The number of animals assessed as experiencing adverse events (x_ADV_) and the total number sampled (n_ADV_) in each study are given.

| **Animal species** | **Operational**  **activity** | **Adverse event type** | ***x_ADV_*** | ***n_ADV_*** | ***p_ADV_*** | **Null** | **Exact test**  ***p*-value** | **Wilson**  **Chi-square statistic** | **Wilson**  ***p*-value** |
| --- | --- | --- | --- | --- | --- | --- | --- | --- | --- |
| Domestic cattle (*Bos taurus*) | Ship transport | Mortality | 742 | 194,216 | 0.004 | 0.01 | 0.000 | 749.13 | 0.000 |
| Domestic sheep (*Ovis aries*) | Ship transport | Mortality | 167 | 9,540 | 0.018 | 0.02 | 0.042 | 3.03 | 0.041 |
| Moose (*Alces alces*) | Helicopter  darting | Mortality | 20 | 2,816 | 0.007 | 0.02 | 0.000 | 23.90 | 0.000 |
| Domestic cattle | Captive bolt  stunning | Non-immediate insensibility | 31 | 304 | 0.102 | 0.05 | 1.000 | 17.29 | 1.000 |
| Gray wolf (*Canis lupus*) | Helicopter  darting | Mortality | 3 | 89 | 0.034 | 0.02 | 0.896 | 0.85 | 0.822***** |
| Western grey kangaroo (*Macropus fuliginosus*) | Captive bolt  euthanasia | Non-immediate insensibility | 1 | 28 | 0.036 | 0.05 | 0.588 | 0.12 | 0.364***** |
| Eastern grey kangaroo (*Macropus giganteus*) | Captive bolt  euthanasia | Non-immediate insensibility | 8 | 21 | 0.381 | 0.05 | 1.000 | 48.42 | 1.000***** |
| Brushtail possum (*Trichosurus vulpecula*) | Kill trapping | Sensible after 3 minutes | 1 | 19 | 0.053 | 0.2 | 0.083 | 2.58 | 0.054***** |
| Brushtail possum | Kill trapping | Sensible after 3 minutes | 1 | 15 | 0.067 | 0.2 | 0.167 | 1.67 | 0.098***** |
| Brushtail possum | Kill trapping | Sensible after 3 minutes | 4 | 11 | 0.364 | 0.2 | 0.950 | 1.84 | 0.913***** |

***** Warning in software R that the underlying assumptions may be violated due to small sample size.

**Table S2. A comparison of the probability that the frequency of observed adverse events (p_ADV_) is less than the threshold (null) value (i.e., ‘innocent until proven guilty’) using the Clopper–Pearson (‘exact’) and Wilson methods.** The source of each study is given in Table 3 in the main text. The number of animals assessed as experiencing adverse events (x_ADV_) and the total number sampled (n_ADV_) in each study are given.

| **Animal species** | **Operational**  **activity** | **Adverse event type** | ***x_ADV_*** | ***n_ADV_*** | ***p_ADV_*** | **Null** | **Exact test**  ***p*-value** | **Wilson**  **Chi-square statistic** | **Wilson**  ***p*-value** |
| --- | --- | --- | --- | --- | --- | --- | --- | --- | --- |
| Domestic cattle (*Bos taurus*) | Ship transport | Mortality | 742 | 194,216 | 0.004 | 0.01 | 0.000 | 749.13 | 0.000 |
| Domestic sheep (*Ovis aries*) | Ship transport | Mortality | 167 | 9,540 | 0.018 | 0.02 | 0.042 | 3.03 | 0.041 |
| Moose (*Alces alces*) | Helicopter  darting | Mortality | 20 | 2,816 | 0.007 | 0.02 | 0.000 | 23.90 | 0.000 |
| Domestic cattle | Captive bolt  stunning | Non-immediate insensibility | 31 | 304 | 0.102 | 0.05 | 1.000 | 17.29 | 1.000 |
| Gray wolf (*Canis lupus*) | Helicopter  darting | Mortality | 3 | 89 | 0.034 | 0.02 | 0.896 | 0.85 | 0.822***** |
| Western grey kangaroo (*Macropus fuliginosus*) | Captive bolt  euthanasia | Non-immediate insensibility | 1 | 28 | 0.036 | 0.05 | 0.588 | 0.12 | 0.364***** |
| Eastern grey kangaroo (*Macropus giganteus*) | Captive bolt  euthanasia | Non-immediate insensibility | 8 | 21 | 0.381 | 0.05 | 1.000 | 48.42 | 1.000***** |
| Brushtail possum (*Trichosurus vulpecula*) | Kill trapping | Sensible after 3 minutes | 1 | 19 | 0.053 | 0.2 | 0.083 | 2.58 | 0.054***** |
| Brushtail possum | Kill trapping | Sensible after 3 minutes | 1 | 15 | 0.067 | 0.2 | 0.167 | 1.67 | 0.098***** |
| Brushtail possum | Kill trapping | Sensible after 3 minutes | 4 | 11 | 0.364 | 0.2 | 0.950 | 1.84 | 0.913***** |

***** Warning in software R that the underlying assumptions may be violated due to small sample size.

Fifth and finally, we used the Wilson method to estimate the one-sided CIs for the six operational and six research applications considered in the main text (Table 5 and Table 6, respectively). There was negligible difference between the size of the one-sided CIs estimated by the exact method (Fig 4) and those estimated by the Wilson method (Fig S4) for the six operational applications in which sample sizes ranged from 296 to 222,293 (Fig S4). Consistent with expectations, the one-sided CIs estimated for the five research activities in which sample sizes ranged from 2 to 23 were smaller when estimated using the Wilson method than those estimated using the exact method (Fig 5, Fig S5). The sizes of the one-sided CIs estimated for the New Zealand fur seal (*Arctocephalus forsteri*) research activity, in which the sample size was 120, were similar for both methods.

**Fig S4. One-sided confidence interval estimates for adverse event frequency estimates from six published animal welfare studies of operational (not research) activities.** Point estimates are shown (grey dots), as are the following confidence intervals: 80% (grey), 90% (green), 95% (blue) and 99% (red). For those studies that used sample sizes of <1000, the confidence intervals are relatively wide for estimating frequencies, whereas for those that used sample sizes of >1000, the confidence intervals are relatively narrow. For study details, see Table 1 in the main text.

**Fig S5. One-sided confidence interval estimates for adverse animal welfare event frequency estimates from six published research trials.** Point estimates are shown (grey dots), as are the following confidence intervals: 80% (grey), 90% (green), 95% (blue) and 99% (red). It is evident that for studies that used small sample sizes, the confidence intervals are wide. For study details, see Table 2 in the main text.

**Conclusion**

The above comparisons confirm that the Clopper–Pearson exact test is indeed more conservative than the Wilson method; however, nominal coverage is always achieved with the exact method but not with the Wilson method. We believe that managers and researchers can be confident in the exact text because its assumptions are less prone to be violated at smaller sample sizes. Importantly, whichever method is used, a sample size of at least several hundred is required in order to obtain a precise estimate of an adverse welfare outcome occurring at low frequency.

**References**

1. Agresti A, Coull BA. Approximate is better than “exact” for interval estimation of binomial proportions. The American Statistician. 1998;52(2):119–26.

2. Wilson EB. Probable inference, the law of succession, and statistical inference. Journal of the American Statistical Association 1927;22:209–12.

3. R Core Team. R: A language and environment for statistical computing. Vienna: R Foundation for Statistical Computing; 2018. Available from: https://www.R-project.org
