# Supplementary material for: How many to sample? Statistical guidelines for monitoring animal welfare outcomes
Source: PLoS One. 2019 Jan 30;14(1):e0211417. doi: 10.1371/journal.pone.0211417 (PMC6353194; doi:10.1371/journal.pone.0211417)
Supplement: S2 Appendix — (DOCX) [file pone.0211417.s002.docx]

**S2 Appendix. Online calculator for determining sample size for animal welfare studies.**

<https://www.proteus.co.nz/resources/bposssc>
